# Effect of sex hormones on human voice physiology: from childhood to senescence

**DOI:** 10.1007/s42000-021-00298-y

**Published:** 2021-05-28

**Authors:** Virginia Zamponi, Rossella Mazzilli, Fernando Mazzilli, Marco Fantini

**Affiliations:** 1 Endocrinology Unit, Department of Clinical and Molecular Medicine, Sant’Andrea Hospital, Sapienza University, Rome, Italy; 2 Head and Neck Oncology Unit, Candiolo Cancer Institute, FPO IRCCS, Candiolo, Turin, Italy

**Keywords:** Sex hormones. Endocrinology, Voice, Communication, Physiology

## Abstract

The aim of the present literature review is to describe the influence of sex hormones on the human voice in physiological conditions. As a secondary sexual organ, the larynx is affected by sex hormones and may change considerably over the lifespan. In the current review, sex hormone-related voice modifications occurring during childhood, puberty, the menstrual cycle, pregnancy and senescence are described. The roles of sex hormones (including gonadotropins, testosterone, estrogen, androstenedione, dehydroepiandrosterone, and dehydroepiandrosterone-sulfate) underlying physiological voice changes are discussed, the main differences between males and females are explained and clinical implications are taken into account.

## Introduction

The voice is one of the most complex and finely developed of human functions, being not only fundamental for social interaction and communication but also for self-recognition and gender identification.

The voice is produced through the interaction of the lungs, the vocal folds and the vocal tract. Functionally, the lungs represent the power supply, while the vocal folds (an oscillator) work as the sound source and the vocal tract acts as an active resonator. Subglottal pressure is primarily responsible for sound pressure level (SPL) perceived as sound intensity. The vibrating vocal folds, by rapidly oscillating and rhythmically contacting each other, generate a fundamental frequency (F0) perceived as sound pitch. Through the vocal tract, the glottal sound is articulated and selectively amplified, obtaining a unique voice timbre [1].

Hormones have a major influence on the voice organ, affecting both the larynx itself and the structures of the vocal tract. Both in physiological conditions and in the case of endocrine disorders, the human voice can be strongly affected with consistent modifications [2]. The larynx, as a secondary sexual organ, is also considerably influenced by sex hormones.

The purpose of the present literature review is to collect and discuss present knowledge about the influence of the sexual hormones on the human voice from childhood to senescence, both in males and in females.

## Elements of voice physiology

The voice organ is composed of three different systems, namely, the respiratory apparatus, the vocal folds and the vocal tract. The vocal folds are multilayer structures composed of a muscle (the vocalis muscle), a ligament (the vocal ligament) and a mucus membrane cover. The planar gap between the vocal folds is called glottis. The vocal tract (or voice resonator) is the anatomical space consisting of the laryngeal vestibule, hypopharynx, oropharynx, oral cavity, nasopharynx and nasal cavities. The coordinated activity of the respiratory system, the vocal folds and the vocal tract results in voice production or phonation. As the airstream generated by the respiratory system passes through the trachea and reaches the adducted vocal folds, subglottal pressure is generated. When the subglottal pressure is high enough, the glottis opens and air passes through. The vocal folds are rapidly pulled together again for the Bernoulli effect and the subglottic pressure rises again. As a consequence, a cyclic vocal fold oscillation is generated and a primary sound with a certain frequency is produced. This sound represents the so-called voice source, which is then acoustically shaped passing through the vocal tract and becoming the acoustic product that we refer to as the human voice [1, 3].

## Childhood

At birth, the human larynx is about one-third the size of an adult larynx. The position of the larynx is higher in the infant, allowing for the epiglottis to stay behind the soft palate, thus permitting the simultaneous sucking and breathing of the newborn. During childhood, the cricoid cartilage gradually descends from the level of the fourth cervical vertebra (C4) and reaches the level of C6 (primary descent). The configuration of the infant’s larynx is quite different from that of the adult, with an omega-shaped epiglottis, an elliptical glottis lumen and a rounded inferior aspect of the cricoid cartilage. The connective and submucosal tissues of the larynx and vocal tract are softer and less fibrous during childhood, with greater pliability and greater swelling response in the event of inflammatory conditions [4].

Sex hormones play an important role from the first period after birth during which a postnatal surge of gonadotropins and sexual hormones is observed. This phase is called “mini-puberty” because of its similarity to the pubertal period [5, 6]. During “mini-puberty”, boys have higher concentrations of circulating testosterone (T) while girls have higher levels of bioavailable estradiol (E2) [7]. Some authors studying the influence of “mini-puberty” on voice production have suggested that the different sex hormone levels may account for distinct sex-related cry melody patterns and the well-known and consistent female advantage in early language development [8–11]. Wermke et al. [7], who analyzed the infant’s crying melody patterns, distinguished between simple (single-arc) melodies and complex (multiple-arc) melodies. The authors found a correlation between the complexity of the cry melody and the E2/sex hormone-binding globulin (SHBG) ratio, thus explaining the vocal gender difference during “mini-puberty”. During childhood, no other evident hormonal changes occur and no relevant sex differences appear in the vocal organ [12].

## Puberty

Puberty is a crucial period of life during which dramatic physiologic and psychologic changes take place. The onset of puberty is preceded by the phenomenon of adrenarche characterized by an increase of androgen secretion by the adrenal glands. The adrenal androgens include dehydroepiandrosterone (DHEA), dehydroepiandrosterone-sulfate (DHEAS), and androstenedione. Adrenarche is involved in the appearance of body odor and axillary and pubic hair. At puberty, the reactivation of the hypothalamic gonadostat and the secretion of gonadotropin releasing-hormone (GnRH) are responsible for gonadotropin secretion (follicle-stimulating hormone (FSH) and luteinizing hormone (LH)) by the pituitary gland, which stimulates the testis and the ovary production of T and E2, respectively. Clinically, the first physiological changes during puberty are the appearance of secondary sex characteristics, in particular, testicular enlargement in males and breast development in females [13].

With puberty, significant sex-related modifications of the voice organ take place, with enlargement and elongation of the larynx, the vocal tract and the vocal folds. Males experience more dramatic changes than females, reaching a mean vocal fold length of 1.6 cm compared to a mean length of 1.0 cm in females and a mean vocal tract length of 16.9 cm compared to 14.1 cm in females. This difference can be explained by the so-called *secondary descent* of the larynx, a male-specific secondary sexual feature occurring during puberty [12]. Newman et al. found androgen receptors both in the cytoplasm and in the nucleus of the vocal fold cells in males. Testosterone is thought to target these receptors during puberty, this resulting in significant elongation and thickening of the vocal folds in males [14].

Functionally, during puberty, voice mutation takes place. With the mutation, voice pitch (F0) drops by about one octave in males, while the F0 of the female voice drops by about 3–4 semitones (Table 1). A parallel change of the vocal range is observed. Voice mutation occurs at a mean age of 12; in males, when testicular growth reaches 20 ml (Tanner stage G5), mutation will start in less than a year. During voice mutation three main stages are observed, as follows [15]:

- Pre-mutation period: voice quality becomes flat and occasionally hoarse and breathy.
- Proper mutational period: uncertain intonation, sudden alterations in pitch, and falsetto-shifts.
- Post-mutational period: gradual maturation of the voice quality, reaching the typical adult voice timbre and range.

**Table 1:** Voice modifications during puberty

	Mean vocal fold lengths	Mean vocal tract length	Mean voice deepening
Females	1.0 cm	14.1 cm	3–4 semitones
Males	1.6 cm	16.9 cm	1 octave

Some authors suggest that voice pitch in adult males correlates with the levels of circulating testosterone, although there is no clear consensus in the literature on the correlation between vocal tract length and testosterone levels [12, 16–18].

## Menstrual cycle

The menstrual cycle is regulated by the increase and decrease of sex hormone levels, in particular, estrogen and progesterone. Estrogen concentrations are reduced during the pre-menstrual phase (days 23–28), the menses period (days 1–4) and the post-menstrual phase (days 5–10). Estrogen levels increase during the late follicular phase (days 7–13) and the highest estrogen levels are observed during the ovulation phase (days 13–15). Progesterone levels increase after ovulation and decrease before menses (days 15–28) [19].

A variety of voice changes have been described across the different phases of the menstrual cycle. Some authors suggest that the best voice quality is observed during the ovulatory phase when estrogen reaches its highest levels [20]. This is not surprising considering that estrogen promotes increased secretion of mucus by the glandular cells above and below the vocal fold edges, resulting in better mucosal viscosity; moreover, high estrogen levels also improve permeability of the blood vessels and capillaries of the vocal folds, with consequent better tissue oxygenation [21]. On the other hand, women often report symptoms of vocal impairment during the pre-menstrual phase. Vocal symptoms, such as decreased vocal efficiency, reduced flexibility, breathiness, fatigued voice, loss of high notes, hoarseness, intonation problems and muffled voice, are known as *dysphonia premenstrualis* [22, 23].

The described voice disorders are, naturally, more crucial among professional voice users such as female singers, as described by Lacina in 1968 [24]. Since singers can experience significant vocal distress in the pre-menstrual period, in the past, many European opera houses used to offer so-called “grace days” to performers who were due to sing during their pre-menstrual period in order to avoid any damage to their vocal folds [25].

Pre-menstrual dysphonia is hypothesized to result from a direct effect of the sex hormones on vocal fold tissues. As demonstrated by Abitbol et al. [21], significant similarities between epithelial smears of the female larynx and vaginal cervix can be observed. Furthermore, several authors have demonstrated the presence of sex hormone receptors (e.g., progesterone and estrogen) on human vocal folds [26–28].

A recent study by Shoffel-Havakuk et al. [29] analyzed the anatomical and functional features of the female vocal folds in different phases of the menstrual cycle. Specifically, examinations were carried out during the early days of the menstrual cycle (low progesterone levels) and during the pre-menstrual phase (high progesterone levels). The authors reported no significant differences in the acoustic analysis, self-assessments, and vocal fold vibratory quality. Significant changes were observed in laryngeal microvascular patterns, with higher congestion during the pre-menstrual days, suggesting that variations in progesterone levels throughout the menstrual cycle may affect laryngeal vascularity.

It is well-known that progesterone also increases epithelial desquamation and reduces the secretions of mucosal glands above and below the vocal fold edges, resulting in mucosal dryness [21]. All these aspects may account for the fluctuation of voice quality in some women during the pre-menstrual period.

## Pregnancy

During pregnancy, psychological, physiological and physical changes occur. High levels of estrogen and progesterone are secreted, while periodic fluctuations are no longer observed. Apart from the menstrual cycle, voice changes also occur across pregnancy, especially during the third trimester. Voice alterations in pregnant women are collectively known as *laryngopathia gravidarum* [30]*.*

The vocal changes observed during pregnancy appear to be an epiphenomenon of hormonal modifications acting both on the respiratory system and the vocal folds [31].

Concerning the respiratory system, both the upper and lower respiratory tracts undergo considerable change during pregnancy. Nasal and nasopharyngeal mucosa may become congested and hyperemic due to hormonal changes occurring in pregnant women. This inflammatory condition promotes mucus hypersecretion, leading to so-called pregnancy-induced rhinitis which affects about 22% of women [32]. As a consequence, oral breathing is more likely to occur in pregnant women, leading to negative effects on voice quality because of dehydrated vocal fold mucosa [33]. During pregnancy, the thorax undergoes anatomic and mechanical changes: the subcostal angle widens from 68° to 103° as the transverse diameter of the chest increases by 2 cm and chest circumference expands by 5–7 cm. With the progression of gestation, the diaphragm rises by 4 cm. As a result, functional residual capacity (FRC), expiratory reserve volume (ERV), residual volume (RV) and total lung capacity (TLC) decrease to various degrees [34, 35]. These aspects explain the constant and significant decrease in maximum phonation time (MPT), especially in the third trimester of pregnancy [31, 36–38].

With regard to vocal fold changes during pregnancy, high levels of progesterone promote reduction of mucus production and an increase in its viscosity, leading to the following: relative dehydration of the larynx; increased epithelial desquamation resulting in a thinning of the vocal fold mucosa; reduced tonicity of the laryngeal muscles; dilatation of the vocal fold microvessels leading to mucosal congestion and inflammatory predisposition [21, 31]. Consequently, symptoms of vocal fatigue and altered vocal quality are often reported by pregnant women during the third trimester. As documented by several researchers, voice self-assessment questionnaires scores (such as the Voice Handicap Index (VHI)) are significantly worse during pregnancy, especially during the third trimester [36–38].

While several authors report similar results in terms of aerodynamic parameter modifications (e.g., MTP) and voice self-assessment questionnaires (e.g., VHI), there is to date no clear consensus in the literature regarding acoustic and perceptive parameter modifications during pregnancy [36–41].

## Senescence

Both men’s and women’s voices undergo changes as part of the aging process. Presbyphonia, also known as age-related dysphonia, refers to aging-related voice disorders and is associated with age-related anatomical modifications of the respiratory system, the vocal tract and the vocal folds [42–45].

Vocal aging is a sexually dimorphic phenomenon, sex-related senescence patterns of the endocrine system possibly explaining the differences between the vocal aging processes of men and women. After menopause, women experience a sudden and dramatic fall in estrogen levels and a relative increase in androgen levels. In contrast, men undergo a progressive decline in androgens, with a consequent relative increase of the estrogen/androgen ratio [46, 47].

Since sex hormone receptors are present in the vocal folds, as demonstrated by several authors, modifications of sex hormone levels during senescence are hypothesized to have a pivotal role in sexually dimorphic modifications of the voice in the elderly [14, 26–28].

As concerns women’s voices, in 1999, Abitbol et al. [21] described a *menopausal vocal syndrome* characterized by lowering of vocal intensity, vocal fatigue and decreased vocal range with a loss of high notes and a loss of timbre in the spoken and singing voice. The latter clinical presentation can be explained by the morphological modifications affecting the larynx after menopause, such as vocal fold thickening, vocal fold edema and increased prominence of the vocal process [43, 45, 48, 49]. The thickening of the vocal folds may explain the increased glottal closure in older women, with a reduced posterior glottal gap, which is also commonly observed in young women [48–50]. As a result, in post-menopausal women, reduced vocal breathiness is observed in comparison to younger women [51]. Another typical vocal change after menopause is a lowering of the F0, with a deeper tone of voice and a virilization of the vocal timbre. Such modification is another consequence of the thickening of the vocal folds due to the relative increase of androgen levels after menopause [21, 49, 52]. On the other hand, after menopause women can experience variable F0 modifications. Since adipose tissue is the main source of estrogen production in post-menopausal women, higher body mass indexes correlate with higher F0 after menopause [53].

As regards men, a progressive thinning of the vocal folds is observed with aging. After the age of 30, serum testosterone levels decrease by 1% per year. The progressive reduction of testosterone levels contributes to sarcopenia in aged men (over 65 years), this also affecting the vocalis muscle [54]. As a consequence, vocal fold bowing and reduced glottal contact occur, with higher perceived breathiness in aged men’s voices compared to those of younger men. Moreover, increased F0 is observed in men’s senescence, with vocal feminization and a clearer tone of voice [16, 52, 55].

In conclusion, in the context of the aging process, men and women experience opposite changes in vocal fold structure and dynamics, speaking tone of voice, and perceived breathiness, as shown in Table 2.

**Table 2:** Voice modifications in senescence

	Sex hormones modification	Vocal folds modifications	Perceptual and acoustic modifications
Women	Fall of estrogen levels, relative increase of androgen levels	Thickening and edema of the vocal folds. Prominence of the vocal process. Increased glottal contact during phonation	Reduced breathiness, lowered F0 with deeper tone of voice and vocal virilization
Men	Progressive decrease of testosterone levels	Thinning and bowing of the vocal folds. Reduced glottal contact during phonation	Increased breathiness, higher F0 with clearer tone of voice and vocal feminization

## Conclusions

The human voice is strongly influenced by sex hormones throughout the lifespan, from childhood to senescence. As a secondary sexual characteristic, voice is deeply affected by sex hormone modifications, with substantial gender differences. The voices of males and females follow a divergent trajectory, reaching its maximum divergence during adolescence and tending to converge again with the aging process.

Vocal changes follow the physiological body modifications during life and reflect the hormonal well-being of a subject, in particular during critical transition processes. Clinicians should pay particular attention to their patients’ voice quality since it mirrors hormonal physiology and can therefore also be a marker of endocrine disorders, with significant implications for physical and psychological health.
